# Biosurfactants and Synthetic Surfactants in Bioelectrochemical Systems: A Mini-Review

**DOI:** 10.3389/fmicb.2020.00358

**Published:** 2020-03-13

**Authors:** Grzegorz Pasternak, Theresia D. Askitosari, Miriam A. Rosenbaum

**Affiliations:** ^1^Laboratory of Microbial Electrochemical Systems, Department of Process Engineering and Technology of Polymer and Carbon Materials, Wrocław University of Science and Technology, Wrocław, Poland; ^2^Laboratory of Microorganism Biotechnology, Faculty of Technobiology, University of Surabaya, Surabaya, Indonesia; ^3^Leibniz Institute for Natural Product Research and Infection Biology – Hans-Knöll-Institute, Jena, Germany; ^4^Faculty of Biological Sciences, Friedrich Schiller University, Jena, Germany

**Keywords:** biosurfactant, surfactant, microbial fuel cell, bioelectrochemistry, anode, cathode, BES

## Abstract

Bioelectrochemical systems (BESs) are ruled by a complex combination of biological and abiotic factors. The interplay of these factors determines the overall efficiency of BES in generating electricity and treating waste. The recent progress in bioelectrochemistry of BESs and electrobiotechnology exposed an important group of compounds, which have a significant contribution to operation and efficiency: surface-active agents, also termed surfactants. Implementation of the interfacial science led to determining several effects of synthetic and natural surfactants on BESs operation. In high pH, these amphiphilic compounds prevent the cathode electrodes from biodeterioration. Through solubilization, their presence leads to increased catabolism of hydrophobic compounds. They interfere with the surface of the electrodes leading to improved biofilm formation, while affecting its microarchitecture and composition. Furthermore, they may act as quorum sensing activators and induce the synthesis of electron shuttles produced by electroactive bacteria. On the other hand, the bioelectrochemical activity can be tailored for new, improved biosurfactant production processes. Herein, the most recent knowledge on the effects of these promising compounds in BESs is discussed.

## Introduction

In recent decades, bioelectrochemical systems (BESs) have undergone dynamic development and raised increasing interest of the scientific community. The term BESs applies to several types of devices, where microorganisms play the crucial role of carrying out various types of electrochemical reactions. In a microbial fuel cell (MFC), organic matter is oxidized by heterotrophic, electroactive anodic bacteria and converted into electric current (Bennetto et al., 1983). Microbial electrolysis cells (MECs) are based on a similar principle but require additional, small portion of electrical energy input to form hydrogen at the cathode (Liu et al., 2005). The potential difference between anode and cathode electrodes is also used for separation of the ionic species in microbial desalination cells (Cao et al., 2009). Finally, the BESs may be also used for producing valuable chemicals. The processes in which the organic and inorganic compounds are produced with the support of electroactive bacteria are referred to as bioelectrosynthesis and electrofermentation (Rabaey and Rozendal, 2010; Moscoviz et al., 2016).

With these potentials, BESs are in transition to a wide variety of applications. The MFC and MEC technology has already been successfully investigated in scale up experiments and pilot scale studies to treat wastewater (Cusick et al., 2011; Ieropoulos et al., 2016; Hiegemann et al., 2019; Rossi et al., 2019a). A great effort has been made to utilize BES as a biosensing devices (Cui et al., 2019) and even MFC-driven autonomous, self-powered biosensor (Pasternak et al., 2017), or robots (Ieropoulos et al., 2010) have been reported. BES can be also used for biological remediation (Li and Yu, 2015; Ramírez-Vargas et al., 2019) and, as previously noted, as water desalination devices. In lab scale, several organic compounds were synthesized as final products of bioelectrochemical reactions such as alcohols (Mayr et al., 2019), acetate, or acetoin (Patil et al., 2015; Förster et al., 2017).

The BESs are based on microorganisms, which are the principal catalysts in these systems. Very often, the microbial biocatalyst is incorporated in a biofilm in BES. The biofilm carrying out the central reactions requires different functionalities and architectures whether the electron transfer is dominated by direct electron transfer via membrane-bound *c*-type cytochromes (Holmes et al., 2004; Lovley et al., 2011) or if it is dependent on indirect diffusion-limited electron transfer with soluble redox mediators (Rabaey et al., 2005; Venkataraman et al., 2011). Bioelectrochemical techniques provide a rich interplay of submolecular, molecular, cellular and, community-based mechanisms, which determine the final efficiency of BES-based processes (Yasri et al., 2019). Therefore, multiple elements affecting BES efficiency are being continuously developed and include engineering of membranes (Dizge et al., 2019; Pasternak et al., 2019), electrodes (Chong et al., 2019), microorganisms (Askitosari et al., 2019), design and modeling approaches as well as development of peripheral systems (Gadkari et al., 2018; de Ramón-Fernández et al., 2019; Tsompanas et al., 2019).

The interface between biotic and abiotic elements in such a complex environment is a matter of a particular importance. Therefore, in this mini-review, we will focus on a group of compounds, which can act at the interface of liquid, solid and gas phases, namely surfactants. Surfactants represent a wide group of amphiphilic compounds, which are widely used in the industry. Examples of widely used surfactant groups include alkyl sulfates (anionic), alkyl ammonium chlorides (cationic), betaines (amphoteric), and ethoxylates (non-ionic). Recently, significant scientific attention has been paid to substitute the chemically synthesized surfactants with biosurfactants, which can be produced by microorganisms *in situ* in various types of bioreactors. Biosurfactant functionalities in living systems have still not been fully understood (Chrzanowski et al., 2012). Examples of the natural roles of biosurfactants include: increasing the surface area and bioavailability of hydrophobic substrates, regulating the attachment, detachment of microorganisms to the surfaces, participating in *quorum sensing* mechanisms, binding of heavy metals, and antimicrobial activity (Ron and Rosenberg, 2001). The above-mentioned functions may therefore have a crucial impact on the BES performance.

**FIGURE 1:**
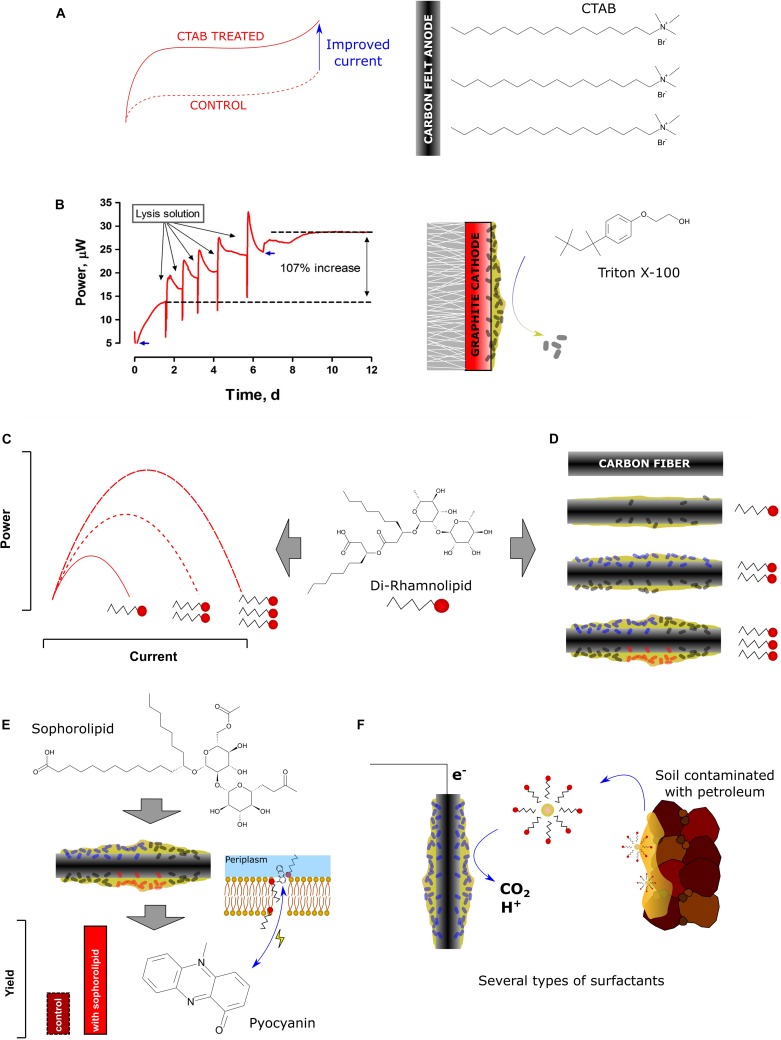
Structures and effects of various synthetic and biological surfactants on bioelectrochemical systems. **(A)** the effect of hexadecyltrimethylammonium bromide (CTAB) carbon felt surface modification on current output (Guo et al., 2014). **(B)** Regeneration of power performance by washing biofouled graphite cathodes [adapted from Pasternak et al. (2016) under CCBY4.0 license]. **(C)** improved power performance (Wen et al., 2010) and **(D)** biofilm structure (Zhang et al., 2017) and composition (Li et al., 2018) by increasing rhamnolipid concentration. **(E)** improved pyocyanin yield and cell membrane permeability through addition of sophorolipid (Shen et al., 2014). **(F)** solubilization of petroleum compounds for bioelectrochemical remediation (Li et al., 2018).

Although synthetic surfactants may have their biological analogs, their functions and applications in biotic and abiotic components of BES may be entirely different. Several types of emerging interactions and applications of synthetic and biosynthesized surfactants in BES were described so far. In high pH, surfactants may prevent the biodeterioration of MFC cathodes (Pasternak et al., 2016). Their presence enhance the bioavailability of hydrophobic substrates (Hwang et al., 2019). They interfere with the surface of the electrodes, which leads to improved biofilm formation (Zhang et al., 2017). Furthermore, they may have synergistic effect with electron shuttles on MFC power performance (Pham et al., 2008). These and other interactions of biosurfactants (Figure 1 and Table 1) along with the possibility of applying BESs for synthesis of these promising and valuable compounds are discussed in this mini-review.

**TABLE 1:** Summary of key effects of surfactants in bioelectrochemical systems.

Parameters	Compounds	Type of application	Positive impact when compared to the control	Possible considerations	References
Current	Rhamnolipid (RL), PCN	Addition to anolyte	Synergistic effect led to reaching EET for Brevibacillus strain	Inhibitory effect above ≥1 mg/L	Pham et al., 2008
	Trehalose	Addition to anolyte	1.83-fold higher	Current decrease above ≥40 mg/L	Cheng et al., 2018
	Sophorolipid	Addition to anolyte	2.6-fold higher	NI	Shen et al., 2014
Power	Tween 80	Addition to anolyte	88% higher	NI	Wen et al., 2011
	Trehalose	Addition to anolyte	5.93-fold higher	Power decrease ≥40 mg/L	Cheng et al., 2018
	Sophorolipid	Addition to anolyte	4-fold higher	NI	Shen et al., 2014
	SDS	Anode modification	20% higher	Power decrease ≥10 mmol	Song et al., 2015
Internal resistance	Trehalose	Addition to anolyte	43% lower	NI	Cheng et al., 2018
	Sophorolipid	Addition to anolyte	40% lower	NI	Shen et al., 2014
	Rhamnolipid	Endogenous overexpression	30% lower	NI	Zheng et al., 2015
Phenazine production	Sophorolipid	Addition to anolyte	1.7-fold lower	NI	Shen et al., 2014
Biofilm density and diversity	Rhamnolipid	Addition to anolyte	2-fold higher thickness, increased coverage and increase of electroactive community by 24.6%	Irreversible, potentially negative changes of the biofilm properties when ≥80 mg/L	Zhang et al., 2017
Cathodic performance recovery	Triton X-100	Washing of the cathode	100% power recovery	Careful handling of lytic solution required	Pasternak et al., 2016
Biodegradation	Tween-80	Addition to catholyte	43.5% higher PCB transformation	NI, possible toxicity of by-products	Yu et al., 2017
Hydrogen production	Rhamnolipid (RL), SDS, SDBS	Addition to anolyte	4-fold higher (for RL)	Type and dose-dependent effect	Zhou et al., 2017

*NI, not investigated.*

## Effects of Surfactants and Biosurfactants on BES Components

### Influence on Electron Transfer Mechanisms

One of the main obstacles for efficient mediated extracellular electron transfer is the barrier function of the bacterial cell walls and membranes, which might not allow the mediators to pass (Lovley, 2006). Surfactants may affect the membrane permeability (Sotirova et al., 2008), which is essential for electron shuttling in the mediated electron transfer (MET), and can lead to enhanced electricity generation (Yong et al., 2013). In that light, a handful of experiments showed that the external addition of surfactants like rhamnolipids (Wen et al., 2010), Tween 80 (Ren et al., 2012), SDS (Song et al., 2015), or Triton X-100 (Oluwaseun, 2015) is efficiently increasing the current generation in MFCs (Figure 1C). Notably, Wen et al. (2011) reported that the addition of the non-ionic surfactant Tween 80 in an air-cathode MFC significantly increased the power generation from 21.5 W/m^3^ (without surfactant) to 187 W/m^3^ (with surfactant). Many studies also showed that the enhancement of membrane permeability efficiently reduced the internal resistance of the BES, and thus increased the electron transfer efficiency (Yu et al., 2011). An experiment conducted by Shen et al. (2014) proved that the addition of sophorolipid effectively reduced the internal resistance (R_int_) by up to ∼ 40%, whereas Cheng et al. (2018) reported the addition of trehalose lipid decreased the R_int_ about 43% in MFC. The same conclusion has also been obtained from a study conducted by Zheng et al. (2015), in which this parameter decreased by ∼30% for a *P. aeruginosa* PA01 strain, which endogenously overexpressed rhamnolipid, when compared to the wildtype strain. These mentioned studies indicate the beneficial influence of surfactants on the acceleration of mediated anodic electron transfer and the reduction of energy consumption due to internal resistances in MFCs (Zhang et al., 2017).

The positive effect has been related mainly to the role of surfactants to form transmembrane channels in the cell membrane. The presence of surfactants enables the reduction of membrane’s resistance, increasing its permeability, accelerating the transport of substances, and enhancing the substrate degradation (Singh et al., 2007). However, the addition of synthetic surfactants to the MFC can also be toxic for the bacteria (Shen et al., 2014). Therefore, a careful study to understand the optimal concentration in the MFC is required. Several studies reported that the effect of surfactants, which were originally produced by bacteria, i.e., biosurfactants such as rhamnolipids, sophorolipids, and trehalose lipids impose less toxicity to the bacteria upon addition. However, while the addition of 20 mg/L trehalose lipid surfactant in an acetate-fed *Rhodococcus pyridinivorans*-inoculated air cathode-MFC resulted in 1.83 times higher currents density and 5.93 times higher power density than the control, the presence of trehalose lipid above this concentration reduced the bacterial metabolism and integrity, which caused a lower electroactivity than in the control (Cheng et al., 2018). The same trend was also found in the experiment conducted by Shen et al. (2014) when sophorolipids have been added to acetate-fed *P. aeruginosa*-inoculated air cathode-MFC. Thus, also in case of biosurfactants, a proper concentration to be applied in MFCs needs to be determined beforehand.

### The Synergistic Interaction Between Phenazines and Surfactants

An important group of redox compounds studied for natural MET are microbial phenazines, which are produced by *Pseudomonas* species, in particular by *Pseudomonas aeruginosa*. Phenazines are synthesized from chorismic acid through phenazine-1-carboxylic acid (PCA) formation, which is further converted into pyocyanin (PYO), 1-hydroxyphenazine (1-HP), and phenazine-carboxyamide (PCN). The synthesis of phenazine is encoded by two homologous operons called operon one and operon two. In *P. aeruginosa* PAO1 and PA14, operon two showed higher activity when compared to operon one in the phenazine synthesis and current production in oxygen-limited BES (Askitosari et al., 2019). In the environment, phenazines act as a virulence factor of *P. aeruginosa* by reducing molecular oxygen into reactive oxygen species, which are toxic for other microbial species (Mentel et al., 2009). On the other hand, in co-culture between *P. aeruginosa* and *Enterobacter aerogenes*, phenazines can promote synergistic interaction between species and be utilized further for electron discharge (Venkataraman et al., 2011; Schmitz and Rosenbaum, 2018). Concerning the interaction between phenazines and surfactants, in *P. aeruginosa*, both the endogenous rhamnolipid surfactant and phenazine synthesis are tightly controlled by a complex genetic regulatory network and they are often co-regulated (Abisado et al., 2018).

According to a study conducted by Pham et al. (2008), the non-electrochemically active bacterium *Brevibacillus* sp. PTH1 was able to generate currents with a combined addition of rhamnolipids up to a concentration of 1 mg/L and phenazine carboxyamide (PCN) produced by *Pseudomonas* sp. CMR12a in the acetate-fed MFC system. The provision of the phenazine alone did not promote electroactivity. It is likely that the surfactant promoted PCN solubility, which enabled the PCN to cross the peptidoglycan layer of gram-positive *Brevibacillus* sp. PTH1. Hence, this bacterium was able to employ PCN as electron shuttle through a synergistic interaction with rhamnolipids, eventually enabling these bacteria to discharge electrons to the anode. In another study, reported by Shen et al. (2014), the addition of 40 mg/L sophorolipids led to the increase of pyocyanin production, current density, and power density (1.7 times, ∼2.6 times, and 4 times, respectively, higher than control) in a *P. aeruginosa*-inoculated air cathode-MFC (Figure 1E). Despite these beneficial effects, the addition of exogenous synthetic or biosurfactants results in higher costs of MFC operation. Therefore, ideally, the biosurfactant should be endogenously produced by the bacteria within the MFC. This should provide physiological and economic advantages for MFC performance. Heterologous production of phenazines to enable electroactivity in *Pseudomonas putida* KT2440 has recently been achieved (Schmitz et al., 2015; Askitosari et al., 2019). This biotechnologically relevant bacterium has also successfully been engineered for rhamnolipid production in the past (Wittgens et al., 2011; Tiso et al., 2017). A next consequential step would now be the combined tailored production of phenazines and surfactants to evaluate the natural synergism in a controlled manner. One of the studies already showed that an endogenously stimulated surfactant production led to increased cell permeability and enhanced biofilm formation, which are both beneficial to trigger enhanced phenazine production, which in turn increases electroactivity (Zheng et al., 2015). More work is to be expected in this cutting-edge field of microbial electrophysiology.

### Influence on Biofilm Formation and Stability

In BESs, the biofilm plays an essential role in electron transfer between the bacterial cells, as well as in cell-electrode interactions. The microbial ability to form the biofilm is essential in harsh, BES conditions, which are dominated by unfavorable environmental conditions, such as low aeration, substrate limitation, and incidental desiccation. The biofilm formation may be induced by the presence of biosurfactants in MFCs. Such effect was observed by several authors so far. A 96-well plate test revealed that overproduction of rhamnolipids resulted in induced biofilm formation by *P. aeruginosa* PAO1– the same strain that authors utilized to determine the effect of biosurfactants on MFC performance (Zheng et al., 2015). This may be explained by the amphiphilic nature of biosurfactants, which facilitate the attachment of the hydrophilic bacterial cell to the hydrophobic substratum. The same effect may thus occur at the electrode surface, in particular carbon-based materials, which are hydrophobic. Overall, the positive effect of microbially produced surfactants in initial colonization of surfaces has been long recognized in other fields of microbiology (e.g., tissue infections, agriculture, or microbial corrosion processes). In contrast, larger amounts of biosurfactants will also destabilize biofilms or cell aggregates and promote more planktonic growth. A more recent study stays in line with this hypotheses and revealed some interesting insights into an effect of rhamnolipids on biofilm adhesion and structure (Zhang et al., 2017). The authors have estimated the biofilm thickness on various levels of rhamnolipid present in the anolyte. The addition of 40, 80, and 120 mg/L of rhamnolipid resulted in the biofilm thickness of 2.03, 6.14, and 4.14 μm, respectively, while only weak attachment was observed when the biosurfactant was not present (Figure 1D). A similar trend was observed, when the biomass of the electroactive community was quantified ranging from 0.42 ± 0.06 mg/m^3^ (control) up to 0.86 ± 0.06 mg/m^3^. Lastly, the microbial community composition of the biofilm was also affected by the presence of rhamnolipid. The MFC supplemented with 40 mg/L had an increased ratio of potential electroactive species such as *Geobacter, Desulfovibrio, Tolumonas*, and *Aeromonas*, reaching 81% when compared to the control (65%). These shifts in microbial community composition may also be related to either tolerance of some groups of bacteria to specific types of surfactants or activation of the quorum sensing mechanisms caused by surfactants which could give a competitive advantages to some of the species.

### Improving the Performance of Anode Electrode

The synthetic surfactants have been recently highlighted as compounds, which can react with the surface of both cathode and anode electrodes in MFCs. Guo et al. (2014) have demonstrated a facile method of increasing the hydrophilic properties of carbon felt electrodes by soaking the electrodes a few minutes in 2 mM cetyltrimethylammonium bromide (CTAB) solution (Figure 1A). Such a strategy resulted in improved bioelectrochemical performance of the anodes (Guo et al., 2014). Similar results were demonstrated by Song et al. (2015) who investigated the surface modification with the use of sodium dodecyl sulfate (SDS). They used a chemically pretreated (with sulfuric and chromic acids) and exofoliated graphite powder treated with surfactant and further with nitric acid. Although the authors have used several treatment steps that could lead to an improved hydrophilicity of the anodic surface (such as acid treatment), the only variable was a surfactant concentration. Modulating the quantity of surfactant between 0 and 20 mM resulted in improving the power production by 20% observed for 5 mM SDS and a decreased lag time, while decreased power output was recorded for 10 and 20 mM concentrations of SDS.

### Improving the Performance of Cathode Electrode

At high concentrations and pH, the surfactants presence may lead to the death of the bacterial cell through disruption of the cell membrane. This process is known as the alkaline lysis and was used by Pasternak et al. (2016) for improving the performance of deteriorated cathodes (Figure 1B). The biofouling problem of cathodes in BES has been widely reported and leads to the significant decrease of MFC performance (Al Lawati et al., 2019; Noori et al., 2019; Rossi et al., 2019b). In the above mentioned study, the authors recorded nearly 91% drop of power, which clearly resulted from the growth of the biofilm at the graphite-based cathode of the ceramic MFCs. The application of a non-ionic surfactant (Triton X-100) in 0.1% concentration along with 0.2 M NaOH heated to 60°C resulted in immediate recovery of the cathodic performance to the levels exceeding 100% and removing the biofilm from the electrode surface, while NaOH heated to the same temperature had no-effect on power regeneration. Such an approach may be an alternative for more commonly proposed mechanical cleaning methods (Rossi et al., 2018) and help to prevent the biofilm recolonization.

## Surfactants and Biosurfactants in BES-Biotechnological Processes

### Biosynthesis

Biosurfactants are currently being produced in industrial scale by several commercial companies. Some examples include BASF Cognis (Germany) producing glycolipids, cellobiose lipids and mannosylerythritol lipids, Boruta-Zachem (Poland) producing surfactin, and Ecover (Belgium) producing sophorolipids. However, their synthesis in aerobic microbial processes is often hampered by energy demand of the bioprocess as well as extensive foaming and resulting problems with biomass retention and product recovery leading to losses of efficiency (Marchant and Banat, 2012). Applying oxygen-limited BESs for synthesis of biosurfactants may overcome some of these challenges. The first report, where the rhamnolipid synthesis was observed in MFCs was published in 2008 and their presence allowed *Brevibacillus* sp. to achieve extracellular electron transfer, as discussed previously (Pham et al., 2008). In another study, Schmitz et al. (2015) have discussed the possibility of using BES along with an engineered *P. putida* strain to synthesize detergents in oxygen-free or oxygen-limiting conditions. This process is enabled through the engineered production of phenazines as redox mediators in non-electroactive *P. putida* to enable anaerobic metabolic reactions. Such an oxygen limitation approach could not only lead to developing foaming-limited biotechnological process based on BESs, but potentially can also result in a higher carbon yield of the products (Schmitz et al., 2015). Furthermore, an engineered strain of *P. aeruginosa* has been used for inducing rhamnolipid production through overexpression of the rhamnosyltransferase gene (rhlA) in a MFC system (Zheng et al., 2015). More recently, the addition of metallic nanoparticles to the cathode electrode resulted in improved power performance and production of biosurfactant (Liu and Vipulanandan, 2017). The authors have recorded significant drop of the surface tension during growth of the biofilm at the anodic potential of −0.3 V and recorded up to 3.14 g/L of crude extracellular lipid products when the Fe-nanoparticles were used. These examples suggest that sustainable, BES-based production of biosurfactants is possible.

### Biodegradation

Surfactants are often used to increase bioavailability of recalcitrant compounds during biodegradation processes (Figure 1F). The study described by Li et al. (2018) showed that the use of surfactants to stimulate the biodegradation of petroleum hydrocarbons in MFCs may affect the taxonomical composition of electroactive biofilms. The authors studied five types of surfactants among which the lecithos – ampholytic surfactant (mainly lecithin) was responsible for the highest power and biodegradation performance. The SDS and β-cyclodextrin (biosurfactant) caused the most selective shifts in bacterial communities. In another study, the presence of Tween 80 resulted in improving the PCB transformation in sediment MFC by 43.5% (Yu et al., 2017). The amphiphilic nature of surfactants was also exploited recently by Hwang et al. (2019), who investigated the biodegradation of bilge water in MFCs. The addition of 100 ppm of anionic SDS resulted in improved power output, which reached 225.3 mW/m^2^, while the use of non-ionic Triton X-100, resulted in two orders of magnitude lower power performance. The use of surfactants may therefore cause several effects (also negative) in biodegradation-oriented bioelectrochemical techniques and the appropriate studies should always precede their utilization.

### Hydrogen Production

Surfactants have also been recognized as methanogenesis inhibiting agents (Jiang et al., 2007). Such a feature may be therefore implemented to improve the hydrogen evolution in MECs. In a recent study, Zhou et al. (2017) have tested SDS, sodium dodecyl benzene sulfonate (SDBS) and rhamnolipids for their influence on hydrogen production. The rhamnolipid addition has boosted the hydrogen yield to 12.90 mg H_2_/g VSS (volatile suspended solids), which was the maximum value when compared to the other surfactants and several times larger when compared to the controls. The authors claimed that rhamnolipids led to the highest acidification of the activated sludge, which was the fuel and possibly improved membrane permeability of the electroactive biofilm cells, leading to improved electrochemical parameters. Contrary results were described by Ren et al. (2012), who investigated the effect of Tween-80 on power performance of MECs, showing that surfactant concentrations up to 20 mg/L had no significant effect on current generation. Furthermore, an adverse effect was observed when its concentration reached 80 mg/L (Ren et al., 2012).

## Concluding Remarks

Surfactants, either synthetic or biological, appear to be a highly reactive group of compounds in terms of their influence on several parameters, which are determining BES performance. Although a relatively rich number of surfactants were examined, only rhamnolipids and sophorolipid were included as representatives of biological surfactants. The recently emerged studies indicate that bio/surfactants may interfere with electron transfer mechanisms, especially electron shuttles such as phenazines, biofilm attachment and survival at the electrodes, as well as biofilm architecture and composition. These advantages make them an ideal target for a novel circular economy approaches in biotechnology, where the waste is being converted into value-added product. The *in-situ* production of surfactants in BESs would offer a great advantage of ensuring an energy-neutral bioprocess modification, which positively affects the BES performance in substrate utilization and energy production through various mechanisms. Considering the range of the effects when different types of surfactants and biosurfactants were investigated in BES, such process, however, would require careful and accurate control in order to avoid the occurrence of negative phenomena such as toxic effects on the electroactive community. Since contrary effects of different surfactants were observed on similar processes, identifying these effects for specific types of surfactants and comparative studies, as well as focus on the potential negative effects will be the main challenge to make use of their advantages in the future. The application of bio/surfactants in BES could finally lead to extended lifetime of the functional MFC elements affected by biofouling, thus leading to their increased utilization toward waste and wastewater treatment.

## Author Contributions

GP conceptualized the scope and outline of the study. GP, TA, and MR performed the literature screening. GP and TA wrote the first draft of the manuscript. MR and GP critically revised the manuscript.

## Conflict of Interest

The authors declare that the research was conducted in the absence of any commercial or financial relationships that could be construed as a potential conflict of interest.
